# Flow Cytometry and AI-Driven Biomarker Discovery in Acute Kidney Injury: From Mechanistic Insights to Translation Applications in Clinical and Austere Environments

**DOI:** 10.3390/ijms27198450

**Published:** 2026-09-22

**Authors:** Shoulei Jiang, Michael Adam Meledeo, Xiaowu Wu, Barbara A. Christy, Timothy S. Horseman

**Affiliations:** Blood and Shock Resuscitation, United States Army Institute of Surgical Research, JBSA, Fort Sam Houston, TX 78234, USA

**Keywords:** acute kidney injury (AKI), flow cytometry, artificial intelligence (AI), machine learning (ML), mitochondrial dysfunction, oxidative stress, biomarkers, precision medicine, endothelial dysfunction, extracellular vesicles (EVs)

## Abstract

Acute kidney injury (AKI) is a complex and multifactorial syndrome characterized by the rapid decline of renal function following ischemic, septic, traumatic, hypoxic, or nephrotoxic insults. Despite advances in critical care, exacerbation of AKI is associated with substantial morbidity, mortality, progression to chronic kidney disease (CKD) and multiorgan dysfunction. Current therapies remain largely supportive and do not directly target the molecular pathways driving AKI, underscoring the need for mechanism-based diagnostics and precision-guided therapeutic strategies. Accumulating evidence supports that AKI is driven by mechanistic crosstalk among cellular injury pathways involving mitochondrial dysfunction, oxidative stress, endothelial damage, immune dysregulation, and regulated cell death. Flow cytometry has emerged as a powerful platform for high-dimensional single-cell and extracellular vesicle analysis, enabling comprehensive assessment of immune cell phenotypes, platelet activation, endothelial alterations, mitochondrial dysfunction, and circulating extracellular vesicles associated with AKI. Advances in artificial intelligence (AI), machine learning and emerging explainable AI (XAI) enhance the ability to identify clinically relevant patterns from complex datasets. This review aims to integrate AKI pathophysiology with flow cytometry-based biomarker discovery and AI-enabled analytical approaches. We discuss translational applications in AKI-relevant clinical settings, including trauma, hemorrhagic shock, sepsis, and radiation-combined injury, with particular emphasis on Prolonged Casualty Care and other austere environments, where rapid, mechanism-based diagnostics are critically needed.

## 1. Introduction

Acute kidney injury (AKI) affects millions of patients annually and remains a major contributor to morbidity, mortality, healthcare utilization, and progression to chronic kidney disease (CKD) worldwide [1]. AKI is characterized by a rapid decline in the glomerular filtration rate (GFR) and accumulation of nitrogenous waste, mostly developing in patients exposed to ischemia–reperfusion injury (IRI), sepsis, severe trauma, nephrotoxins, hemorrhagic shock, and radiation exposure [1]. The mortality associated with severe AKI remains high, often exceeding 40% in intensive care units (ICU) with a high risk of progression to CKD and end-stage renal disease (ESRD) [2,3].

The current clinical standard for diagnosing and staging AKI is based on the Kidney Disease: Improving Global Outcomes (KDIGO) criteria, which define AKI by an increase in serum creatinine (SCr) of ≥0.3 mg/dL within 48 h or to ≥1.5 times baseline within the preceding 7 days, and/or by reduced urine output (oliguria), and classify disease severity into three stages [4]. However, these functional markers are limited by delayed detection and poor sensitivity during the early stages of kidney injury.

Emerging evidence suggests that AKI should be viewed as a highly complex, systems-level syndrome involving endothelial dysfunction, mitochondrial bioenergetic failure, immune dysregulation, platelet activation, and inter-organ crosstalk [5].

This review summarizes current advances in flow cytometry- and AI-based approaches for mechanism-driven biomarker discovery in AKI, providing a framework for earlier diagnosis, prognostic stratification, and precision medicine in AKI.

The literature for this review was identified through comprehensive searches of PubMed, Web of Science, and Embase databases for peer-reviewed studies published through July 2026. Search terms combined ‘acute kidney injury’ or ‘renal failure’ with ‘flow cytometry’, ‘spectral cytometry’, ‘extracellular vesicles’, ‘artificial intelligence’, ‘machine learning’, and ‘prolonged casualty care’. Selection prioritized seminal mechanistic discoveries, high-parameter cytometry methodologies, and translational studies evaluating biomarker performance.

## 2. Pathophysiological Mechanisms of AKI

AKI is complex and driven by overlapping pathways; understanding its pathophysiology is important for biomarker discovery. Despite their distinct etiologies, ischemic, septic, and nephrotoxic AKI converge on rapid mitochondrial bioenergetic failure. This drives oxidative stress, downstream cellular injury, and the release of damage-associated molecular patterns (DAMPs), particularly oxidized mitochondrial DNA (mtDNA), into the cytosol and systemic circulation.

During severe AKI, persistent mitochondrial dysfunction and ATP depletion drive regulated cell death, including apoptosis, necrosis, necroptosis and ferroptosis. Among these, iron-dependent ferroptosis has emerged as a major contributor to tubular injury in both ischemic and nephrotoxic AKI (e.g., cisplatin or folic acid-induced injury) [6,7]].

Concurrently, endothelial dysfunction disrupts the renal microvasculature, promoting ischemia, inflammation, and impaired tissue repair. The earliest event in vascular injury is the degradation of the endothelial glycocalyx (including syndecan-1, heparan sulfate, and hyaluronic acid) lining the luminal surface of blood vessels. As a result, early microvascular endothelial collapse not only deprives the renal parenchyma of oxygen but also shifts the local microenvironment into a maladaptive procoagulant state, rendering the kidney highly vulnerable to secondary ischemic insults.

Endothelial injury rapidly promotes platelet adhesion and activation, initiating thromboinflammation that links vascular dysfunction with innate immune activation. Activated platelets adhere to the damaged endothelium and release inflammatory mediators. This, in turn, amplifies thromboinflammation by promoting microvascular thrombosis, leukocyte recruitment, and leukocyte extravasation [8]. Consequently, localized microvascular occlusion further restricts medullary blood flow, exacerbating hypoxia within the highly vulnerable, low-oxygen environment of the renal medulla [9].

Building upon platelet-driven thromboinflammatory processes, dysregulated innate immune responses further exacerbate renal injury during AKI. Activated platelets form aggregates with circulating neutrophils, promoting platelet–leukocyte crosstalk that facilitates neutrophil recruitment and activation within the renal microvasculature. This interaction stimulates the formation of Neutrophil Extracellular Traps (NETs) [10].

Although NETs evolved as an essential host defense mechanism for trapping pathogens during infection, excessive NET formation in sterile ischemic AKI becomes maladaptive. Consequently, sustained recruitment and activation of innate immune cells transform localized cellular stress into a self-perpetuating inflammatory cascade, worsening renal tissue injury.

Collectively, the interconnected pathways described above illustrate the multifactorial nature of AKI pathogenesis (Figure 1).

Although these mechanisms are shared across many forms of AKI, their relative contribution varies substantially among different etiologies. This biological heterogeneity has led to the concept of AKI phenotypes and mechanistic endotypes, which can be distinguished using biomarker-driven approaches.

Integrating multiple mechanistic biomarkers provides a foundation for accurate disease characterization, mechanistic patient stratification, and precision medicine. This mechanistic framework underpins the following discussion of AKI phenotypes, biomarker discovery, and the emerging roles of flow cytometry and AI.

## 3. Clinical AKI Phenotypes and Mechanistic Endotypes

Clinical phenotypes describe the precipitating clinical syndrome (e.g., IRI, sepsis, or nephrotoxic exposure), whereas mechanistic endotypes refer to the dominant biological pathways driving renal injury. Different AKI phenotypes exhibit distinct molecular, cellular, and immunologic signatures, providing a framework for mechanism-based biomarker discovery and patient stratification. The major AKI phenotypes, their clinical settings, dominant mechanistic endotypes, representative biomarker signatures, and potential applications of multiparametric flow cytometry are summarized in Table 1.

### Implications for Biomarker-Guided Precision Medicine

The recognition that AKI comprises multiple biologically distinct and potentially overlapping phenotypes has important implications for biomarker-guided precision medicine. Rather than relying solely on serum creatinine and urine output, future diagnostic strategies will integrate molecular biomarkers, flow cytometric signatures, imaging data, and clinical variables to define mechanistically meaningful endotypes. Such multidimensional approaches may improve patient stratification and facilitate more personalized therapeutic interventions.

The phenotypic diversity of AKI highlights the limitations of relying solely on functional markers such as serum creatinine and urine output. Because distinct AKI subtypes exhibit unique cellular and molecular signatures, considerable efforts have focused on identifying biomarkers capable of detecting injury earlier, differentiating underlying mechanisms, and improving risk stratification. However, conventional functional markers may provide limited insight into the specific biological pathways driving renal injury.

The diverse mechanisms described above generate distinct cellular and molecular biomarkers that reflect specific injury pathways. However, because no individual biomarker adequately captures AKI complexity, multiplexed approaches capable of simultaneously measuring multiple mechanistic processes have become increasingly important, making multiparametric flow cytometry a powerful platform for mechanism-based biomarker discovery and patient stratification. This approach enables concurrent assessment of immune-cell populations, endothelial and platelet-derived extracellular vesicles, mitochondrial stress, oxidative injury, and cell-death pathways.

Although many biomarkers—including inflammatory mediators, endothelial activation markers, oxidative stress indicators, and extracellular vesicles—are shared across multiple AKI etiologies, individual biomarkers rarely demonstrate disease specificity. Rather than relying on a single marker, high-dimensional flow cytometry enables simultaneous characterization of multiple cellular phenotypes and functional states. Integration of these multidimensional biomarker signatures with appropriately validated computational analysis may facilitate identification of distinct mechanistic AKI endotypes and improve etiologic classification and risk prediction.

Representative established and emerging biomarkers reflecting these diverse AKI pathways are summarized in Table 2, highlighting their biological targets, detection methods, and potential clinical applications.

## 4. Flow Cytometry Applications in AKI Biomarker Discovery

Flow cytometry enables rapid, multiparametric analysis of cellular phenotypes, activation states, functional responses, and extracellular vesicles at single-cell resolution using small sample volumes. Unlike bulk analytical methods such as ELISAs, proteomics, or transcriptomics, flow cytometry preserves cellular heterogeneity by directly interrogating individual cells and their functional states, allowing identification of rare cell populations and dynamic biological responses that are frequently obscured by population-averaged assays. In this way, unique mechanistic information for biomarker discovery and longitudinal disease monitoring is achieved.

### 4.1. Immune Cell Profiling and Endothelial Assessment

Flow cytometry simultaneously identifies immune cell subsets, activation states, and functional phenotypes at single-cell resolution. For example, monocyte and macrophage populations play critical roles in both injury and repair. Flow cytometric analysis of CD14 and CD16 distinguishes classical, intermediate, and nonclassical monocyte subsets with distinct inflammatory functions. Additionally, flow cytometric assessment of neutrophil activation markers such as CD11b and CD66b enables evaluation of adhesion, degranulation, oxidative burst, and NET formation. Characterization of T lymphocyte populations has further revealed important roles for adaptive immunity in AKI progression and recovery, with identification of regulatory T cells (Tregs), typically identified by CD4, CD25, and FoxP3 expression.

Multiparametric flow cytometry also enables direct characterization of circulating endothelial cells (CECs) and endothelial-derived extracellular vesicles (EVs using markers such as CD31, CD144 (VE-cadherin), and CD146, together with Annexin V for phosphatidylserine-positive vesicles.

Beyond circulating blood leukocytes, flow cytometry has proven uniquely valuable for the non-invasive interrogation of urine and single-cell renal tissue digests in AKI. Flow-cytometric quantification of shed urinary renal tubular epithelial cells (RTECs; CD13^+^, CD10^+^, E-cadherin^+^) and injured podocytes provides direct diagnostic resolution of renal parenchymal injury, outperforming conventional urine microscopy in distinguishing intrinsic acute tubular necrosis from transient functional azotemia [38]. Furthermore, high-parameter multiparametric flow cytometry and single-cell profiling of dissociated murine kidneys following ischemia–reperfusion injury demonstrated that tissue-resident macrophages survive severe ischemic insults and dynamically reprogram toward a developmental, reparative state (characterized by F4/80, CD11b, CSF1R, and Ly6C dynamics) to orchestrate renal repair and tubular regeneration [39]. These studies illustrate how high-dimensional cytometry directly bridges localized intra-renal cellular pathophysiology with non-invasive biomarker monitoring.

### 4.2. Platelet Cytometry and Mitochondrial Evaluation

Flow cytometry enables direct assessment of platelet phenotype, activation, and procoagulant activity via surface markers such as CD41, CD42b, CD61 CD62P (P-selectin), αIIbβ3, and phosphatidylserine (PS) [15].

Functional evaluation of mitochondrial dysfunction can be assessed by flow cytometric probes such as Tetramethyl rhodamine Ethyl Ester (TMRE) and JC-1 to permit quantitative evaluation of mitochondrial membrane potential (ΔΨm).

Furthermore, MitoSOX Red can be used to selectively measure mitochondrial superoxide production, whereas CellRox reagents assess total intracellular oxidative stress.

### 4.3. Extracellular Vesicles

Extracellular vesicles (EVs) reflect ongoing cellular injury and intercellular communication. Despite the diagnostic potential of EVs, their small size, heterogeneous cellular origins, and diverse molecular cargo make them difficult to characterize using conventional biochemical assays or bulk assays. Flow cytometry provides an opportunity to characterize phenotypically distinct EV populations; however, several technical limitations must be considered.

Flow cytometry also faces analytical challenges for the detection of EVs, including detection thresholds for small particles, interference from background noise, potential lipoprotein contamination, and challenges in sample preparation. Small EVs may approach or fall below the detection limits of conventional flow cytometers, while high particle concentrations can result in coincident or “swarm” detection. In addition, lipoproteins, protein aggregates, and other non-vesicular particles may overlap with EVs in size and generate nonspecific signals, complicating accurate EV identification and quantification. Pre-analytical variables, including sample collection, processing, storage, staining, and instrument settings, can further contribute to inter-study variability. The Minimal Information for Studies of Extracellular Vesicles 2023 (MISEV2023) guidelines emphasize standardized EV separation and characterization, appropriate controls, calibration, and transparent reporting to improve rigor and reproducibility [40]. For flow cytometric EV analysis, appropriate dilution, negative and staining controls, and complementary approaches such as detergent sensitivity can help distinguish membrane-bound EVs from background particles. These methodological considerations remain important barriers to the standardization and clinical translation of EV-based biomarkers in AKI.

Representative applications are summarized in Table 3.

### 4.4. High-Parameter and Spectral Cytometry

Conventional multicolor flow cytometry has substantially advanced AKI research but remains constrained by spectral overlaps, limited multiplexing capacity, and cellular autofluorescence. High-parameter and spectral flow cytometry overcome these limitations by capturing the complete emission spectrum of each fluorophore, enabling simultaneous analysis of 30 or more biomarkers from a single micro-volume blood or urine sample. For AKI research, this enhanced multiplexing capability allows comprehensive characterization of immune activation, endothelial dysfunction, platelet responses, extracellular vesicles, and mitochondrial injury within a single assay, providing a systems-level view of disease biology [44,45].

## 5. Multi-Omics Integration, Artificial Intelligence (AI), and Computational Cytometry

### 5.1. Multi-Omics Integration

Beyond individual biomarker discovery and validation, multi-omics integration represents the next frontier in AKI diagnostics and therapeutics. By integrating transcriptomics, proteomics, metabolomics, and spatial omics datasets, these approaches provide a comprehensive, unbiased view of the complex pathophysiology of renal injury and facilitate the discovery of novel molecular targets.

While early sub-phenotype classification relied primarily on circulating protein biomarkers, modern precision nephrology increasingly integrates multi-layered omics to map localized spatial and cellular microenvironments. For example, Sun et al. combined single-nucleus RNA sequencing (snRNA-seq) with spatial transcriptomics in human kidney biopsies, demonstrating how mitochondrial-associated metabolic pathways directly dictate local immune cell infiltration and regional injury severity [46]. Furthermore, multi-omics has expanded our systemic understanding of the disease; the integration of metagenomics and metabolomics has highlighted the ‘gut-kidney axis’ as a major pathological driver, revealing how gut-derived metabolites modulate renal MAPK signaling pathways during nephrotoxic insult [47]. When integrated with high-dimensional flow cytometry and AI-driven computational analysis, these complementary molecular platforms provide an increasingly comprehensive framework for mechanistic AKI subphenotyping and precision medicine.

### 5.2. AI-Driven Biomarker Discovery

The rapid expansion of spectral flow cytometry and multi-omics technologies has generated high-dimensional datasets that exceed the analytical capacity of conventional statistical methods and manual gating. Consequently, AI and machine learning have become essential tools for extracting clinically meaningful biological patterns from these complex datasets. AI encompasses computational approaches capable of identifying complex patterns and relationships within large biological and clinical datasets, whereas machine learning (ML), a major subset of AI, enables algorithms to learn from data and improve predictive performance without explicit programming. Deep learning (DL), an advanced form of ML based on multilayer artificial neural networks, is particularly effective for analyzing high-dimensional flow cytometry, multi-omics, medical imaging, and electronic health record (EHR) data. Together, these approaches provide the computational foundation for biomarker discovery, patient risk prediction, predictive modeling and precision medicine in AKI.

Advanced flow cytometry platforms, including spectral and imaging cytometry, play a central role in AI-driven biomarker discovery by generating high-dimensional single-cell datasets (often >30–40 parameters per cell) that far exceed the analytical capacity of traditional manual gating [48]. By integrating flow cytometric measurements with multi-omics and electronic health record data, AI-driven approaches enable biomarker discovery, patient stratification, subphenotype identification, predictive modeling, and precision medicine in AKI [49,50]. The conceptual framework for integrating flow cytometric biomarkers with machine learning and artificial intelligence approaches is illustrated in Figure 2.

The clinical value of AI-assisted AKI prediction has already been demonstrated in several landmark studies. For example, Tomašev et al. developed a deep learning model using EHR data that predicted AKI up to 48 h earlier than conventional clinical diagnosis, highlighting the potential of AI for early risk stratification and intervention [51]. In parallel, unsupervised machine learning approaches have identified biologically distinct AKI subphenotypes associated with differential inflammatory profiles, clinical outcomes, and therapeutic responses [52]. These advances have progressed from retrospective risk prediction to real-time clinical decision support through automated electronic alerts, exemplified by the Nephrotoxic Injury Negated by Just-in-Time Action (NINJA) program, which significantly reduced the incidence and severity of nephrotoxic AKI in hospitalized children [53]. Together, these studies demonstrate the potential of AI to translate complex biological and clinical datasets into actionable decision support.

### 5.3. Unsupervised Learning

Conventional manual gating relies on a priori knowledge, which biases discovery toward known cell types. Unsupervised dimensionality reduction techniques, such as t-SNE (t-Distributed Stochastic Neighbor Embedding) and UMAP (Uniform Manifold Approximation and Projection), allow researchers to visualize millions of cells in two-dimensional space while preserving complex multiparametric relationships [54]. When paired with automated clustering algorithms like FlowSOM or PhenoGraph, these approaches can identify candidate cellular populations and phenotypes that may be overlooked by conventional gating.

These approaches have been widely adopted for high-dimensional flow cytometry and single-cell datasets, enabling data-driven identification and characterization of immune and inflammatory cell populations associated with AKI progression. For example, multiparametric flow cytometry has identified alterations in circulating myeloid populations, including CD14^+^CD16^+^ intermediate monocytes, following cardiac surgery, illustrating the potential of multidimensional cellular profiling to identify signatures associated with postoperative organ dysfunction and AKI [41]. However, direct evidence specifically integrating unsupervised computational cytometry with AKI outcomes remains limited. Beyond cellular biomarker discovery, unsupervised machine learning strategies have identified clinically relevant AKI sub-phenotypes with distinct inflammatory signatures, outcomes, and therapeutic responses [55,56], demonstrating how computational approaches can translate multidimensional biological data toward precision medicine.

While end-to-end clinical validation in human AKI remains an active area of investigation, proof-of-concept is demonstrated in related acute inflammatory and critical care paradigms. For example, computational clustering (such as FlowSOM and Phenograph applied to high-parameter spectral cytometry) has successfully delineated novel immunophenotypes—such as dysregulated monocyte subsets and CD4^+^/CD8^+^ T-cell exhaustion states—that predict sepsis-induced organ dysfunction and secondary AKI progression where conventional gating failed. Furthermore, unsupervised single-cell and mass cytometry pipelines have recently identified distinct myeloid activation trajectories that distinguish transient from persistent renal dysfunction, providing a blueprint for AI-driven immunophenotyping in nephrology.

### 5.4. Predictive Modeling

To translate biomarker discoveries into clinical decision support, supervised machine learning models integrate flow cytometry, multi-omics, laboratory measurements and longitudinal electronic health records (EHRs) data to predict AKI onset, progression, and clinical outcomes [51,57].

Predictive modeling is among the most mature clinical applications of AI in AKI and supports early diagnosis, risk stratification, and outcome prediction.

Static Risk Prediction: Algorithms such as Random Forests and XGBoost (eXtreme Gradient Boosting) are well-suited for structured, point-in-time data. These models identify complex nonlinear interactions among clinical variables and biomarkers, thereby improving prediction accuracy compared with conventional statistical approaches [58].

Dynamic temporal Forecasting: Deep learning architectures, particularly Recurrent Neural Networks (RNNs) and Long Short-Term Memory (LSTM) networks, are designed to handle time-series data. By continuously incorporating serial physiological measurements, laboratory values, urine output and potentially flow cytometric biomarkers, these models dynamically update the probability of AKI progression over time [59]. Deep learning models trained on longitudinal EHR data have demonstrated the ability to predict AKI up to 48 h before conventional clinical diagnosis [51]. Future integration of serial flow cytometry and multi-omics data may further improve predictive performance by incorporating dynamic mechanistic biomarkers into longitudinal prediction models.

Although current predictive models rely primarily on EHR and routine laboratory data, incorporating flow cytometry-derived biomarkers and complementary multi-omics profiles may enhance biological characterization, improve patient stratification, and support more individualized therapeutic strategies.

### 5.5. Explainable AI (XAI) and Federated Learning

Despite the remarkable predictive performance of modern AI models, limited interpretability remains one of the principal barriers to routine clinical implementation. Explainable AI approaches, such as SHAP (SHapley Additive exPlanations), improve transparency by identifying the variables that contribute most to individual predictions, thereby increasing clinicians’ confidence in AI-assisted decision-making [60,61,62]. Complementing explainable AI, federated learning (FL) enables AI models to be trained collaboratively across multiple hospitals without sharing raw patient data, preserving patient privacy while improving model robustness and generalizability [63]. While facing challenges in data heterogeneity and lack of workflow standardization, recent studies have demonstrated the feasibility of FL for multi-center AKI prediction across geographically distributed healthcare systems [64]. These approaches are particularly attractive for multicenter AKI studies because they enable collaborative model development while preserving patient privacy and improving model generalizability across diverse healthcare systems.

Table 4 summarizes representative artificial intelligence and machine learning methodologies currently applied to AKI biomarker discovery, prediction, patient stratification, and computational cytometry.

Collectively, advances in AI and computational cytometry are transforming high-dimensional biological and clinical datasets into actionable tools for AKI diagnosis, patient stratification, and clinical decision support. As these technologies undergo prospective validation, they are expected to play an increasingly important role in trauma, prolonged casualty care (PCC), and other austere settings.

## 6. Translational and Clinical Applications of Flow Cytometry and AI in Acute Kidney Injury

The mechanistic pathways, biomarkers, flow cytometry applications, and AI-driven analytical approaches discussed throughout this review have broad translational relevance across diverse causes of AKI. Their value is particularly evident in prolonged casualty care (PCC), where delayed evacuation, hemorrhagic shock, ischemia–reperfusion injury, sepsis, radiation exposure, and limited diagnostic resources complicate early AKI diagnosis and management. In these austere conditions, integrating rapid flow cytometric biomarker assessment with AI-assisted clinical decision support may improve patient triage, risk stratification and timely therapeutic intervention [14].

### 6.1. Trauma, Hemorrhagic Shock, and AKI

As discussed in Section 2 and Section 5, traumatic hemorrhage rapidly induces mitochondrial dysfunction, endothelial injury, platelet activation, and immune dysregulation that precede overt renal dysfunction. Flow cytometry enables simultaneous assessment of these interconnected mechanisms, while AI-assisted analysis may facilitate rapid patient stratification during PCC. Hemorrhagic shock remains one of the leading causes of trauma-associated AKI in both civilian and military populations. Massive blood loss and tissue injury trigger systemic endothelial dysfunction (SHINE syndrome), coagulopathy, platelet activation, inflammation, and oxidative stress, resulting in renal hypoperfusion and disruption of the endothelial glycocalyx [15]. These early pathophysiological changes promote the development of AKI well before conventional functional markers, such as serum creatinine, become abnormal.

Importantly, many of these cellular responses also occur following severe trauma in the absence of AKI. Therefore, interpretation of individual biomarkers should always be considered within the broader clinical context, and integration of multiple mechanistic biomarkers is likely to provide greater diagnostic specificity than reliance on any single marker.

Flow cytometry enables integrated assessment of these interconnected injury responses from a single blood sample. When integrated with AI-driven predictive models, these high-dimensional biomarker signatures may improve early AKI recognition, individualized resuscitation strategies, and prognostic assessment in trauma patients.

### 6.2. Prolonged Casualty Care (PCC)

Modern military operations increasingly require prolonged casualty care (PCC), in which evacuation may be delayed for 24–72 h while advanced laboratory testing and renal replacement therapy remain unavailable [71]. During this prolonged period, AKI represents a major secondary complication resulting from trauma, hemorrhagic shock, prolonged hypoperfusion, and nephrotoxic medications [47]. Conventional diagnostics based on serial serum creatinine measurements are often impractical under these austere conditions [48].

To bridge this capability gap, ruggedized, portable flow cytometry platforms engineered for low-volume whole-blood analysis can perform rapid, point-of-care profiling of early, sub-clinical organ injury. Utilizing capillary fingerstick blood offers a highly practical, minimally invasive alternative to traditional venous blood draws, bypassing the cumbersome and time-consuming nature of conventional laboratory-based kidney assays in austere environments [49]. By employing targeted fluorophore-conjugated antibody panels, these devices can simultaneously evaluate cellular and molecular cascades, including immune and endothelial activation, platelet dysregulation, and mitochondrial oxidative stress.

When integrated with AI-assisted clinical decision-support systems, these multidimensional biomarkers may support individualized fluid resuscitation, optimize limited medical resources, and improve evacuation prioritization during PCC.

### 6.3. Crush Injury, Rhabdomyolysis, and Myoglobin-Induced Nephrotoxicity

As discussed in Section 2, oxidative stress, mitochondrial dysfunction, and regulated cell death are central mechanisms underlying pigment-induced AKI following crush injury and rhabdomyolysis. Flow cytometry enables simultaneous assessment of these interconnected pathways, providing mechanistic information beyond conventional laboratory biomarkers. Physical trauma in austere environments frequently involves crush injuries and structural collapses, triggering severe rhabdomyolysis [44]. The subsequent massive lysis of skeletal muscle cells releases myoglobin, iron, and damage-associated molecular patterns into the circulation, causing direct pigment-induced tubular toxicity, intense ROS generation via iron-mediated Fenton reactions, and intra-tubular cast formation [16]. This process could be exacerbated by systemic hypovolemia and the potential use of common field medications, which disable renal autoregulatory mechanisms and accelerate acute tubular necrosis [50]. Although conventional laboratory tests identify established muscle injury, they provide limited information regarding the evolving biological mechanisms that contribute to AKI. Rapid, field-deployable detection of highly oxidized cellular states and early tubular stress markers can dictate aggressive, early alkalinization of urine and targeted fluid therapy before irreversible necrosis occurs.

### 6.4. Radiation Combined Injury (RCI)

Radiation combined injury (RCI) refers to exposure to ionizing radiation occurring concurrently with traumatic injury, thermal burns, hemorrhage, or other physical insults. RCI is considered a major concern following nuclear accidents, radiological terrorism, or military nuclear events because combined injuries produce substantially greater morbidity and mortality than either radiation exposure or trauma alone.

The severity of AKI depends on factors including radiation quality (e.g., gamma or X-ray exposure), absorbed dose, time after exposure, and the nature of the accompanying traumatic injury. Ionizing radiation triggers immediate intracellular damage, inducing DNA double-strand breaks (DSBs), widespread endothelial apoptosis, oxidative stress, and systemic inflammation, all of which contribute to AKI development [72]. When coupled with trauma, these overlapping pathological mechanisms synergize compound tissue hypoxia, microvascular collapse, and systemic immune dysregulation, substantially increasing susceptibility to acute kidney injury (AKI). Because multiple injury pathways evolve simultaneously, radiation-combined injury is particularly well suited for high-dimensional flow cytometry integrated with AI-assisted computational analysis [52].

Dissecting this biological complexity requires diagnostic modalities capable of high-throughput, multi-endpoint cellular analysis. Flow cytometry offers a robust methodology to address this requirement by enabling the simultaneous characterization of DNA damage kinetics, systemic cell-cycle checkpoints, and apoptotic pathways. Within this diagnostic framework, the quantification of phosphorylated histone H2AX (γH2AX) serves as a sensitive, gold-standard metric for immediate, radiation-induced DNA double-strand breaks [19]. This assessment can be paired dynamically with Annexin V staining to monitor membrane phosphatidylserine externalization, capturing early apoptotic cascades before macroscopic cell loss occurs. Furthermore, the platform’s multi-color capabilities allow for the concurrent profiling of hematopoietic and endothelial injury, mapped through leukocyte depletion kinetics, platelet dysregulation, and the release of microvascular endothelial-derived extracellular vesicles.

Translating these heterogeneous, high-dimensional flow cytometry datasets into actionable clinical insights requires the integration of advanced machine-learning algorithms. In austere, mass-casualty scenarios, where centralized laboratory infrastructure may be unavailable, AI-assisted biodosimetry can synthesize these multi-organ cellular signatures into predictive injury models. By establishing objective, molecular-level triage thresholds, this integrated computational paradigm facilitates rapid injury stratification and guides the targeted, timely deployment of radioprotective medical countermeasures (MCMs) before irreversible multi-organ injury develops.

### 6.5. Future Direction: Label-Free Morphology-Based Flow Cytometric Assessment

The integrated framework presented throughout this review also creates opportunities for next-generation biomarker discovery beyond conventional fluorescence-based immunophenotyping. While fluorescence-based immunophenotyping remains the definitive standard for flow cytometric biomarker analysis, future diagnostic advances may benefit from intrinsic cellular characteristics. During acute kidney injury (AKI) and systemic trauma, circulating immune cells, platelets, endothelial cells, and renal-derived extracellular vesicles undergo dynamic morphological remodeling. These physical transformations—encompassing cellular activation, swelling, cytoskeletal reorganization, degranulation, apoptosis, and membrane blebbing—alter how cells scatter light in flow cytometry. These intrinsic cytometric parameters, namely forward scatter (FSC) (reflecting relative cell size) and side scatter (SSC) (reflecting internal granularity and complexity), can capture these structural alterations in real time. Because these scatter parameters are automatically acquired during standard cytometric acquisition, they represent rapid, complementary biological data that requires zero additional antibody reagents, complex staining protocols, or specialized sample preparation.

Although morphology-derived cytometric features have been explored in other injury models, their application to AKI remains largely unexplored. AI-assisted analysis of intrinsic scatter characteristics represents a promising future direction for computational flow cytometry. Future studies should investigate whether morphology-derived features extracted from FSC and SSC provide complementary information to established molecular and functional biomarkers and whether their integration with AI-driven analytical frameworks improves mechanistic characterization and early AKI risk assessment.

Integrating these intrinsic scatter profiles with high-dimensional cytometric datasets and machine learning algorithms may enable the development of multi-parameter, morphology-based biomarker signatures that complement conventional fluorescence-based measurements. As miniaturized cytometric instrumentation and computational pipelines continue to evolve, morphological profiling could become a component of precision medicine frameworks for early AKI detection.

Future computational approaches may integrate morphology-derived features with immunophenotypic, functional, multi-omics, imaging, and clinical datasets to generate multidimensional biomarker signatures. Such an integrated analytical framework may improve injury phenotyping, patient stratification, prognostic prediction, and therapeutic monitoring. As computational cytometry continues to evolve, label-free morphology-derived features may become a valuable complement to established biomarker panels in precision medicine for AKI.

Collectively, integrating mechanistic biomarkers, high-dimensional flow cytometry, multi-omics, and AI-driven computational analysis establishes a comprehensive precision medicine framework for AKI. Beyond improving early detection, these multidimensional biomarkers also create opportunities for biomarker-guided therapeutic intervention, which are discussed in the following section.

## 7. Therapeutic Opportunities and Precision Interventions

The clinical utility of flow cytometric biomarker profiling is fully realized when it is directly coupled with precision therapeutic interventions. Rather than relying on empiric, one-size-fits-all treatments, future biomarker-guided approaches may enable clinicians to deploy targeted pharmacological agents guided by real-time cellular signatures. For example, the early detection of distinct ferroptosis biomarkers—such as flow cytometric quantification of robust lipid peroxidation (via BODIPY 581/591 C11 staining) and intracellular iron accumulation—can serve as the specific clinical trigger for administering ferroptosis inhibitors (e.g., ferrostatin-1 or liproxstatin-1) before massive tubular necrosis occurs [53].

Similarly, real-time cytometric profiling of hyperactivated innate immune subsets and elevated neutrophil extracellular trap (NET) formation could guide the precise deployment of immunomodulatory agents or targeted DAMP scavengers [73]. In austere environments and Prolonged Casualty Care (PCC) scenarios, this biomarker-to-therapeutics linkage ensures that limited, high-value pharmacological assets are administered only when a casualty’s specific, molecular-level AKI endotype dictates their use, thereby maximizing both therapeutic efficacy and resource conservation.

Despite advances in understanding AKI mechanisms, current treatments remain largely supportive (e.g., fluid management, avoidance of nephrotoxins, and timing of Renal Replacement Therapy—RRT) [74,75]. The goal of biomarker discovery extends beyond improving diagnosis to enabling biomarker-guided precision medicine. Mechanism-based biomarkers identified through flow cytometry, multi-omics profiling, and AI-driven analytical approaches have the potential to identify patients with distinct biological endotypes, allowing therapeutic interventions to be tailored to the dominant pathways driving kidney injury. Consequently, emerging therapies increasingly target specific mechanisms such as mitochondrial dysfunction, ferroptosis, endothelial injury, and maladaptive repair rather than treating AKI as a single homogeneous syndrome.

### 7.1. Targeting Bioenergetics and Mitophagy

Mitochondria-targeted antioxidants such as MitoQ and elamipretide (SS-31) selectively partition into the inner mitochondrial membrane to directly neutralize ROS, preserve ATP production, and protect cardiolipin from oxidation [60]. Furthermore, modulation of mitophagy represents a highly promising frontier. Pharmacological inhibition of the fission protein dynamin-related protein 1 (Drp1) with agents like Mdivi-1 prevents excessive mitochondrial fragmentation [61]. Conversely, enhancing the PINK1/Parkin-mediated mitophagy pathway helps rapidly clear damaged, ROS-leaking mitochondria, preventing downstream activation of necroptosis and limiting the release of DAMPs [62].

### 7.2. Ferroptosis Inhibition and Microvascular Protection

Emerging discoveries have shifted focus toward ferroptosis inhibition. Agents like ferrostatin-1 or liproxstatin-1 are actively investigated to block iron-dependent lipid peroxidation, which plays a major role in ischemic and nephrotoxic AKI [6]. Additionally, targeting NETs with DNase therapy or specific neutrophil elastase inhibitors offers a strategy to mitigate microvascular occlusion [76]. In the realm of vascular protection, targeted delivery of Angiopoietin-1 or Tie-2 agonists aims to reconstitute the endothelial glycocalyx and halt microvascular leakage.

### 7.3. Cellular Senescence and Maladaptive Repair

Beyond cellular death pathways, cellular senescence has emerged as a key driver of maladaptive repair and the subsequent transition from AKI to CKD. Following acute injury, a subset of renal cells enters a state of permanent cell-cycle arrest while remaining metabolically active, secreting a pro-inflammatory and pro-fibrotic cocktail known as the senescence-associated secretory phenotype (SASP).

Targeting senescent cells with senolytics (agents that selectively induce apoptosis in senescent cells) represents a promising therapeutic approach. Recent single-cell RNA sequencing has identified distinct senescent macrophage populations during acute muscle injury-induced AKI, suggesting that their clearance may mitigate downstream tissue damage [77].

However, the timing of senolytic therapy is critical [78]. Guerrero et al. demonstrated that early administration of the senolytic combination Dasatinib and Quercetin (D + Q) during acute experimental nephropathy exacerbated tubular injury and failed to rescue protective renal Klotho levels [79]. These findings suggest that transient early-phase cellular senescence plays an essential role in tissue repair, cautioning against premature senolytic therapy during acute AKI.

Translating senolytic therapies (e.g., Dasatinib + Quercetin, Navitoclax) from preclinical AKI models to clinical trials involves distinct methodological hurdles [79]. First, defining the precise therapeutic window is paramount: while acute senescence is essential for early wound healing and limiting fibrosis, persistent senescence drives chronic maladaptive repair and transition to CKD. Consequently, prophylactic or hyper-acute administration can exacerbate tubular injury. Second, dosing schedules must utilize intermittent ‘hit-and-run’ dosing rather than continuous exposure to clear senescent cells while minimizing off-target cytotoxicity and hematologic toxicities. Finally, trials require validated surrogate biomarkers of response—such as longitudinal monitoring of urinary senescence-associated secretory phenotype (SASP) components (e.g., IL-6, TGF-beta, MMPs) and circulating senescence-associated microRNAs—to stratify patients and confirm target engagement.

### 7.4. Precision Clinical Trials

Innovative delivery mechanisms, such as nanomedicine-based drug delivery, allow for the targeted release of therapeutics directly to the proximal tubule, minimizing systemic side effects [74]. Ultimately, integrating multi-marker flow cytometry panels with AI risk-stratification will enable “predictive enrichment” in clinical trials. Rather than giving a ferroptosis inhibitor to all AKI patients, AI diagnostics will ensure the drug is administered exclusively to the patient subpopulation actively exhibiting a ferroptotic and highly oxidative molecular signature.

## 8. Challenges and Future Perspectives

Translating multidimensional, flow cytometry-based biomarker approaches into routine clinical practice presents several hurdles. Chief among these is the lack of assay standardization, particularly regarding pre-analytical variables in extracellular vesicle isolation (e.g., adhering to MISEV guidelines) [36]. Instrument harmonization across different hospital networks and strict regulatory barriers for clearing “black-box” AI-assisted diagnostic software also remain significant bottlenecks.

Additional challenges include the cost and accessibility of high-parameter flow cytometry platforms, the need for specialized technical expertise, and integration of multidimensional datasets into routine clinical workflows.

Large, prospective, multicenter validation studies are essential to establish standardized clinical cut-offs across diverse populations. Continued integration of multi-omics technologies with real-time cytometry and computational analytics will continuously refine prognostic accuracy. Future directions include the use of patient “digital twins” for therapeutic simulation and longitudinal immune monitoring to guide precision critical care medicine.

## 9. Conclusions

Acute kidney injury is a multifactorial syndrome driven by mitochondrial dysfunction, oxidative stress, endothelial injury, immune dysregulation, and microvascular collapse. Flow cytometry, particularly when integrated with AI-driven computational approaches, provides a versatile platform for the multidimensional characterization of these processes and may facilitate mechanism-based diagnosis, prognostication, and patient stratification.

Several important challenges remain before these approaches can be broadly translated into clinical practice. First, flow-cytometric biomarker panels require validation in larger, diverse, and multicenter AKI cohorts. Second, standardized sample processing, marker selection, gating strategies, and analytical workflows are needed to improve reproducibility across laboratories. Third, the temporal dynamics of cellular biomarkers must be better defined across AKI onset, progression, and recovery. Fourth, AI-based models require prospective validation, external testing, and improved interpretability before routine clinical implementation.

Future research should therefore prioritize prospective longitudinal studies integrating flow cytometry with established kidney injury biomarkers, clinical variables, and other multi-omics data; development of standardized and simplified flow-cytometric panels; external validation of interpretable AI models; and adaptation of portable analytical platforms for point-of-care and resource-limited settings. Continued interdisciplinary collaboration between nephrologists, immunologists, military trauma surgeons, and computational scientists is vital to translate these biological insights into actionable precision medicine platforms for both state-of-the-art ICUs and austere environments.

## Figures and Tables

**Figure 1:**
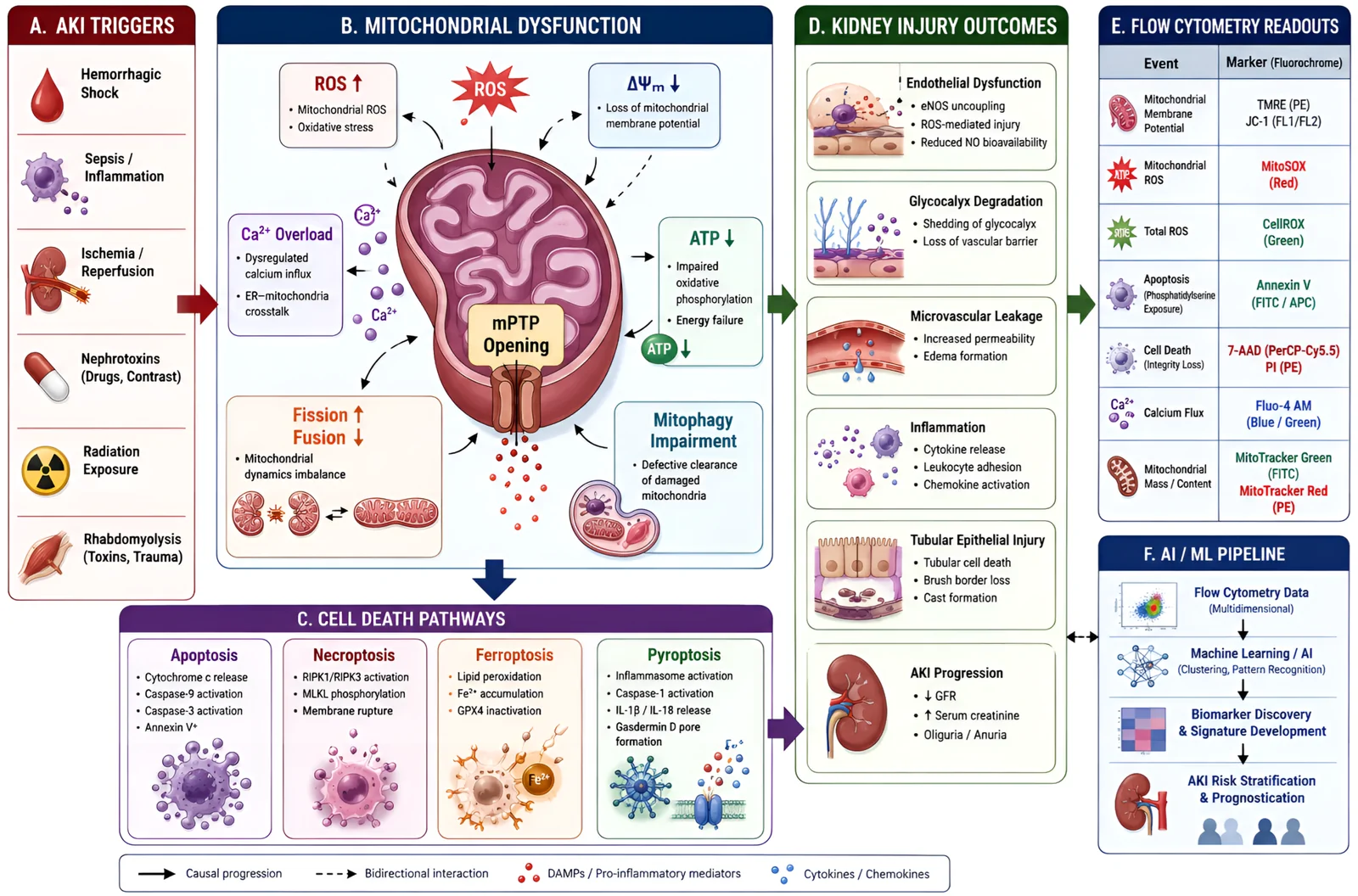
Mitochondrial dysfunction as a central driver of acute kidney injury (AKI) pathogenesis and flow cytometry-based biomarker discovery. (**A**) AKI can be initiated by diverse insults, including hemorrhagic shock, sepsis and inflammation, ischemia–reperfusion injury, nephrotoxic drugs, radiation exposure, and rhabdomyolysis. (**B**) These stressors induce mitochondrial dysfunction characterized by excessive reactive oxygen species (ROS) production, calcium overload, mitochondrial membrane depolarization (∆ψm loss), ATP depletion, impaired mitophagy, altered mitochondrial fission/fusion dynamics, and mitochondrial permeability transition pore (mPTP) opening. (**C**) Mitochondrial injury subsequently activates multiple regulated cell death pathways, including apoptosis, necroptosis, ferroptosis, and pyroptosis, (**D**) leading to tubular epithelial injury, endothelial dysfunction, microvascular damage, inflammation, and loss of renal function. (**E**) Flow cytometry enables quantitative assessment of mitochondrial and cellular injury through biomarkers such as TMRE or JC-1 (mitochondrial membrane potential), MitoSOX (mitochondrial ROS), Annexin V (apoptosis), 7-aminoactinomycin D (7-AAD; membrane integrity), calcium-sensitive probes, and mitochondrial mass indicators. (**F**) Integration of these high-dimensional flow cytometry datasets with artificial intelligence (AI) and machine learning (ML) approaches may facilitate biomarker discovery, patient stratification, early AKI prediction, and precision medicine applications. Solid and dashed arrows indicate casual progression and interactions, respectively; red and blue dots represent DAMPs/Proinflammatory mediators and cytokines/chemokines, respectively. Upward and downward arrows indicate increase and decreases, respectively.

**Figure 2:**
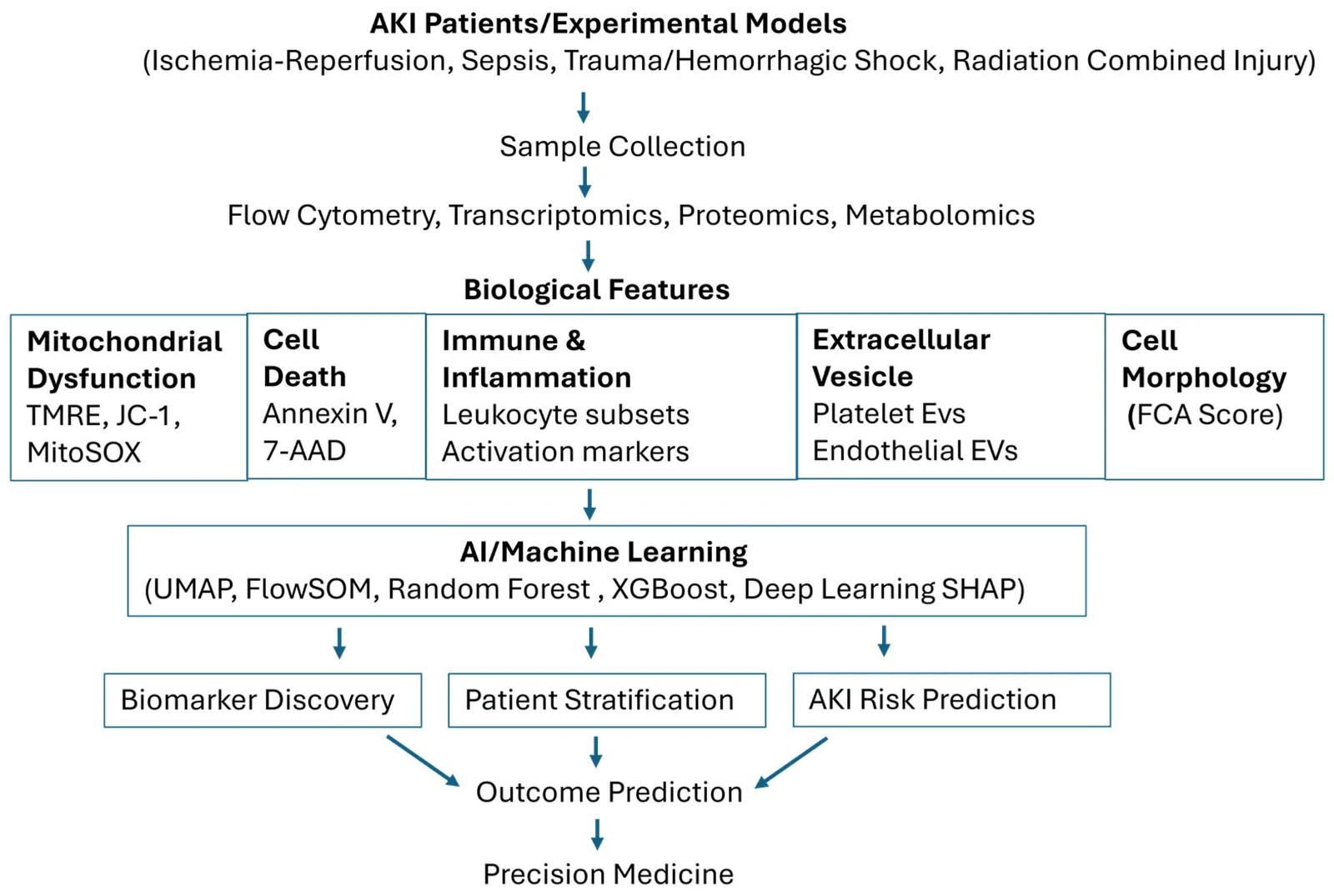
Integrated AI-driven workflow for flow cytometry and multi-omics biomarker discovery in AKI. Biological samples obtained from AKI patients or experimental models are analyzed using flow cytometry and complementary omics platforms, including transcriptomics, proteomics, and metabolomics. Features reflecting mitochondrial dysfunction, cell death, immune activation, extracellular vesicle (EV) release, and cellular morphology (including FCA score—based metrics) are integrated using machine learning approaches such as UMAP, FlowSOM, Random Forest, XGBoost, SHAP and deep learning models. These analyses support biomarker discovery, patient stratification, AKI risk prediction, outcome prediction, and precision medicine applications.

**Table 1:** Acute kidney injury (AKI) phenotypes, dominant pathophysiological mechanisms, representative biomarker signatures, and potential applications of multiparametric flow cytometry. AKI encompasses multiple mechanistic endotypes characterized by distinct biological pathways and molecular profiles. Representative biomarkers associated with ischemia–reperfusion injury, sepsis-associated AKI, rhabdomyolysis-induced AKI, nephrotoxic AKI, and radiation-combined injury are summarized. These biomarker signatures may facilitate mechanistic stratification, early diagnosis, risk prediction, and precision medicine approaches.

AKI Phenotype	Common Clinical Context	Dominant Mechanistic Endotypes	Key Biological Features	Representative Biomarker Signature	Potential Contribution of Multiparametric Flow Cytometry
Ischemia–Reperfusion Injury (IRI) [11,12]	Cardiac surgery, transplantation, shock	Mitochondrial dysfunction, ROS generation, endothelial injury	Ischemia followed by reperfusion promotes mitochondrial injury, ROS generation, tubular-cell stress, and apoptotic signaling. These changes may precede increases in serum creatinine.	NGAL, KIM-1, L-FABP, TIMP-2/IGFBP7, mtDNA	Characterization of mitochondrial membrane potential, ROS generation, and apoptosis or cell-death signaling to assess early cellular stress and tubular injury.
Sepsis-Associated AKI [13]	Septic shock, critical illness	Inflammation, endothelial dysfunction, glycocalyx degradation, thromboinflammation	Renal dysfunction may occur despite relatively preserved global renal perfusion. Biological heterogeneity reflects variable inflammatory, endothelial, and microvascular injury patterns.	Ang-2, Syndecan-1, IL-6, IL-18, suPAR, endothelial EVs	Resolution of activated leukocyte subsets, inflammatory phenotypes, and endothelial or platelet-derived EV populations that may be obscured in bulk plasma measurements.
Trauma & Hemorrhagic Shock [14,15]	Severe physical trauma, major blood loss, combat casualties	Systemic hypovolemia, endotheliopathy of trauma (SHINE), glycocalyx shedding, platelet–leukocyte aggregation	AKI is frequently multifactorial, reflecting the combined effects of tissue hypoperfusion, endothelial activation, glycocalyx shedding, platelet–leukocyte interactions, and systemic physiologic stress.	Syndecan-1, Angiopoietin-2, soluble thrombomodulin, Platelet-derived EVs (PEVs), Neutrophil CD64	Integrated profiling of platelet and leukocyte activation, platelet–leukocyte aggregates, endothelial and platelet-derived EVs, and vascular-immune disruption.
Rhabdomyolysis-Induced AKI [16]	Crush injury, burns, trauma	Myoglobin toxicity, oxidative stress, ferroptosis	Myoglobin and other intracellular contents contribute to oxidative injury, tubular obstruction, iron dysregulation, and mitochondrial damage.	CK, myoglobin, oxidative stress markers, mtDNA	Functional assessment of intracellular iron accumulation, lipid peroxidation, ROS generation, mitochondrial dysfunction, and cellular death pathways.
Nephrotoxic AKI [17,18]	Cisplatin, contrast agents, drugs	Tubular toxicity, apoptosis, mitochondrial injury	Toxic exposures may produce direct tubular-cell injury and stress responses before overt changes in serum creatinine.	NGAL, KIM-1, TIMP-2/IGFBP7, DKK3, miRNAs	Assessment of mitochondrial stress, oxidative injury, apoptosis, and other cellular signatures that may help distinguish direct toxicity from secondary ischemic or hemodynamic stress.
Radiation Combined Injury (RCI) [19]	Radiation plus trauma/burns	DNA damage, oxidative stress, endothelial dysfunction	Injury may arise from direct radiation effects and synergistic interactions with trauma, burns, hemorrhage, and systemic inflammatory responses.	γH2AX, TMRE/JC-1, MitoSox, EMPs, Platelet-derived EVs (PEVs)	Concurrent assessment of DNA-damage signaling, ROS generation, mitochondrial impairment, apoptosis, and vascular injury to characterize overlapping injury pathways.

**Table 2:** Representative Established and Emerging Biomarkers in Acute Kidney Injury. Biomarkers are categorized according to their principal pathophysiological pathways, including pre-injury stress, tubular epithelial injury, cell cycle arrest, mitochondrial dysfunction, oxidative stress, inflammation, endothelial injury, cell death, and extracellular vesicle signaling. Representative biomarkers, detection methods, and potential clinical applications are summarized.

Biomarker Category	Specific Biomarkers	Primary Pathophysiological Target	Optimal Detection Method	Clinical Utility
Pre-Injury Stress	DKK3, suPAR [13,20]	Chronic tubular stress and baseline immune activation	Pre-Injury Stress	Identifying patients at high risk of developing postoperative or contrast-induced AKI
Tubular Epithelial Injury	NGAL [21], KIM-1, IL-18 [22]	Proximal tubule structural damage and inflammation	ELISA, Point-of-care assays	Early detection of subclinical AKI hours prior to creatinine rise
Cell Cycle Arrest	TIMP-2, IGFBP7 [23,24]	G1 cell cycle arrest secondary to ischemic/toxic stress	Immunoassay (NephroCheck)	Predicting risk of progression to severe AKI within 12–24 h
Mitochondrial & Oxidative	mtDNA [25,26], TMRE [27], JC-1 [28], MitoSOX [29,30], CellRox [30]	Disrupted bioenergetics, mitochondrial fragmentation	Flow Cytometry, PCR	Early detection of mitochondrial dysfunction, oxidative stress, and injury progression
Endothelial & Vascular	CD31+/CD144+ EMPs [31], Syndecan-1 [32,33], Angiopoietin-2 [34]	Glycocalyx degradation, microvascular permeability	Flow Cytometry, ELISA	Assessing vascular integrity, risk of fluid overload, and shock
Immune & Inflammatory Response	CD14/CD16 monocytes [5,35], CD66b neutrophils [10]	Leukocyte activation and systemic inflammation	Flow Cytometry	Mechanistic stratification and immune profiling
Cellular Apoptosis & Death	Annexin V, 7-AD, PI [6,7]	Apoptosis, necrosis, and regulated cell death pathways	Flow Cytometry	Mechanistic assessment of injury progression
Extracellular Vesicles (EVs)	Annexin V + EVs, platelet EVs, endothelial EVs [31,36,37]	Cell–cell communication and vascular injury	Flow Cytometry	Early detection, prognostic monitoring, and assessment of vascular injury

**Table 3:** Representative Published Applications of Flow Cytometry in acute kidney injury (AKI) Biomarker Research. The table summarizes selected experimental and clinical studies that have employed flow cytometry to investigate cellular and molecular mechanisms associated with AKI. Included studies encompass immune cell profiling, assessment of apoptosis and regulated cell death, mitochondrial dysfunction, oxidative stress, endothelial injury, platelet activation, and extracellular vesicle characterization. Key findings, biomarkers, flow cytometric targets, and translational implications are highlighted to illustrate the expanding role of flow cytometry in AKI pathophysiology, biomarker discovery, and precision medicine.

Author/Year	Model/Context	Key Biomarkers Assessed	Major Findings	Ref.
Mossanen et al. (2020)	Human; Cardiac Surgery	CD14, CD16 (Monocyte subsets)	An increase in the proportion of CD14++CD16+ intermediate monocytes was strongly associated with the development of post-operative AKI.	[41]
Jansen et al. (2017)	Human; Ischemic AKI	Myeloperoxidase-DNA complexes (NETs)	Neutrophil Extracellular Traps (NETs) were detected in the plasma of patients with established AKI, and their levels correlated with the degree of renal dysfunction.	[42]
Burger et al. (2015)	Mouse; IRI	Exosome markers (CD63, CD9)	Exosomes derived from human endothelial colony-forming cells protected against AKI by delivering pro-angiogenic microRNAs, highlighting a therapeutic role for EVs.	[37]
Gründler K et al. (2014)	Human; Severe Sepsis	TMRE (Platelet mitochondrial membrane potential)	Platelet mitochondrial depolarization correlated with sepsis severity and organ dysfunction.	[27]
Ma et al. (2021)	Human; Cardiac Surgery-Associated AKI	CD31, CD144 (Endothelial Microparticles)	Elevated endothelial microparticles independently predicted postoperative AKI development.	[31]
Wagner et al. (2025)	Human; Clinical AKI & AKD	Urinary renal tubular epithelial cells (RTECs; CD13, CD10, E-cadherin)	Demonstrated flow-cytometric quantification of shed urinary tubular epithelial cells directly correlates with renal parenchymal injury severity, distinguishing intrinsic AKI from pre-renal states.	[38]
Lever et al. (2019)	Murine; Ischemia–Reperfusion Injury (IRI)	Kidney-resident macrophages (F4/80, CD11b, CSF1R, Ly6C)	Multiparametric flow cytometry and single-cell profiling demonstrated that resident macrophages survive injury and reprogram toward a developmental state to facilitate renal repair.	[39]
Dott et al. (2024)	Human whole blood	High-dimensional spectral flow cytometry	Demonstrated standardized high-dimensional immune phenotyping applicable to translational biomarker studies	[43]

**Table 4:** Representative Artificial Intelligence and Machine Learning Approaches for AKI Biomarker Discovery, Phenotyping, and Clinical Decision Support.

AI Method	Data Input	Application in AKI	Key Value	Ref.
Clinical Predictive Modeling	EHR, laboratory data	Early prediction of AKI before serum creatinine elevation	Enables proactive risk stratification and intervention	[51,65]
Phenotype Identification	Multi-omics, flow cytometry	Identification of biologically distinct AKI subphenotypes	Supports precision medicine and targeted therapies	[55]
Clinical Decision Support Systems	EHR, pharmacy data	Automated AKI and nephrotoxin alerts	Facilitates real-time clinical decision-making	[66]
UMAP/t-SNE	High-dimensional flow cytometry	Cellular visualization and subset discovery	Identify rare populations and cellular heterogeneity	[67]
FlowSOM/PhenoGraph	High-dimensional cytometry and single-cell datasets	Automated cellular phenotyping	Reduces operator bias and reveals novel immune signatures	[68,69]
Random Forest/XGBoost	Clinical, biomarker, and multi-omics datasets	AKI prediction and biomarker prioritization	Captures nonlinear relationships and ranks key predictors	[51]
LSTM/RNN	Longitudinal EHR and laboratory data	Temporal prediction of AKI progression and recovery	Model dynamic disease trajectories over time	[65]
Autoencoders/Neural Networks	Multi-omics and flow cytometry datasets	Feature extraction and pattern recognition	Detects latent biological signatures and complex interactions	[70]

Abbreviations: AKI, acute kidney injury; EHR, electronic health record; UMAP, Uniform Manifold Approximation and Projection; t-SNE, t-distributed stochastic neighbor embedding; LSTM, long short-term memory; RNN, recurrent neural network.

## Data Availability

No new data were created or analyzed in this review. Data sharing is not applicable to this article.

## Abbreviations

The following abbreviations are used in this manuscript:

AKI	Acute kidney injury
CKD	Chronic kidney disease
KDIGO	Kidney Disease: Improving Global Outcomes
GFR	Glomerular filtration rate
mPTP	Mitochondrial permeability transition pore
SCr	Serum creatinine
EHRs	Electronic health records
IRI	Ischemia–reperfusion injury
PCC	Prolonged casualty care
RCI	Radiation-combined injury
Ang-2	Angiopoietin-2
NETs	Neutrophil Extracellular Traps
DAMPs	Damage-associated molecular patterns
mtDNA	Mitochondrial DNA
KIM-1	Kidney injury molecule-1
NGAL	Neutrophil gelatinase-associated lipocalin
TIMP-2	Tissue inhibitor of metalloproteinases-2
suPAR	Soluble urokinase plasminogen activator receptor
EMPs	Endothelial-derived microparticles
VCAM-1	Vascular cell adhesion molecule-1
EVs	Extracellular vesicles
ROS	Reactive oxygen species
L-FABP	Liver-type fatty acid-binding protein
TIMP-2	Metalloproteinases-2
IGFBP7	Insulin-like growth factor-binding protein 7
TMRE	Tetramethylrhodamine Ethyl Ester
AI	Artificial intelligence
ML	Machine learning
XAI	Explainable AI
FL	Federated learning
SHAP	SHapley Additive exPlanations
UMAP	Uniform Manifold Approximation and Projection
t-SNE	t-distributed stochastic neighbor embedding
LSTM	Long short-term memory
RNN	Recurrent neural network
